# Supplementary material for: Feasibility of Bio–Coagulation Dewatering Followed by Bio–Oxidation Process for Treating Swine Wastewater
Source: Int J Environ Res Public Health. 2023 Feb 8;20(4):2990. doi: 10.3390/ijerph20042990 (PMC9966416; doi:10.3390/ijerph20042990)
Supplement: Supplementary file 1 [file ijerph-20-02990-s001.zip › ijerph-2183383-supplementary.pdf]

International Journal of
Environmental Research and Public Health

MDPI

Supplementary Files

Feasibility of Bio–Coagulation Dewatering Followed by Bio–Oxidation Process for Treating Swine Wastewater

Dejin Zhang [1,†], Weicheng Han [1,†], Yujun Zhou [2], Cheng Yan [1], Dianzhan Wang [1], Jianru Liang [1] and Lixiang Zhou [1,*]

[1] Department of Environmental Engineering, College of Resources and Environmental Sciences, Nanjing Agricultural University, Nanjing 210095, China
[2] Jiangsu Key Laboratory of Chemical Pollution Control and Resources Reuse, School of Environmental and Biological Engineering, Nanjing University of Science and Technology, Nanjing 210094, China
* Correspondence: lxzhou@njau.edu.cn
† These authors contributed equally to this work.

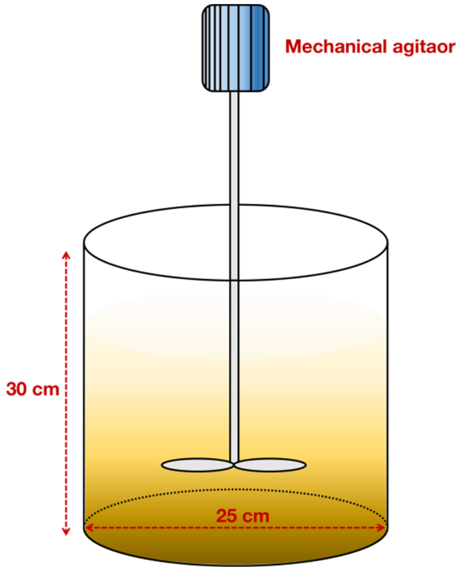

Figure S1. Schematic diagram of reactor used in pilot-scale dewatering experiment.
